# Preoperative imaging for hyperparathyroidism often takes upper parathyroid adenomas for lower adenomas

**DOI:** 10.1038/s41598-023-32707-0

**Published:** 2023-05-09

**Authors:** Annick Van den Bruel, Jacqueline Bijnens, Helena Van Haecke, Vincent Vander Poorten, Catherine Dick, Tom Vauterin, Frank De Geeter

**Affiliations:** 1 Internal Medicine, Endocrinology, AZ Sint-Jan, Bruges, Belgium; 2 Otorhinolaryngology-Head and Neck Surgery, AZ Sint Maarten, Mechelen, Belgium; 3 AZ Sint-Jan, Bruges, Belgium; 4 Otorhinolaryngology-Head and Neck Surgery, University Hospitals Leuven, Leuven, Belgium; 5 Section Head and Neck Oncology, Department of Oncology, KU Leuven, Leuven, Belgium; 6 Otorhinolaryngology-Head and Neck Surgery, AZ Sint-Jan, Bruges, Belgium; 7 Nuclear Medicine, AZ Sint-Jan, Bruges, Belgium

**Keywords:** Three-dimensional imaging, Anatomy, Endocrinology

## Abstract

We retrospectively evaluated how accurately preoperative imaging localizes parathyroid adenoma in superior versus inferior parathyroids. Over 6 years, 104 patients with primary hyperparathyroidism underwent parathyroid surgery in a single centre. Of these, 103 underwent ultrasound, 97 [^99m^Tc]pertechnetate/MIBI SPECT/CT and 30 [^18^F]fluorocholine (FCH) PET/CT. One patient with a unilateral double adenoma was excluded from the analysis. Surgical findings with histopathologic confirmation of adenoma were used as the standard. Ultrasound misjudged 5 of 48 detected lower adenomas as upper, but 14 of 29 upper adenomas as lower (error rate 10 vs 48%, p = 0.0002). The corresponding error rates for [^99m^Tc]pertechnetate/MIBI SPECT/CT were 3 versus 55% (p = 0.000014), and for [^18^F]FCH PET/CT 17 versus 36% (p = 0.26). Our results suggest that about half of the superior parathyroid adenomas which are detected, are erroneously assigned to the inferior position by both ultrasound and SPECT/CT imaging whereas the opposite mistake is significantly less frequent with ultrasound and SPECT/CT.

## Introduction

A superior parathyroid adenoma is defined as a mass adjoining the superior pole of the right thyroid lobe, while an inferior parathyroid adenoma is defined as a mass located posteriorly and inferiorly to the inferior pole of the thyroid lobe^1^. This classification refers to the embryologic origin of the parathyroids. The superior parathyroid glands originate from the fourth branchial pouch along with the ultimobranchial body, while the inferior parathyroid glands and the thymus originate from the third branchial pouch. While the anatomic location of superior parathyroid glands is relatively constant on the posterior aspect of the thyroid (in over 90% of the population), the longer migration tract of inferior parathyroid glands along with the thymus explains their variable position and frequent presentation in ectopic (mediastinal) locations. They are located at the level of the inferior pole of the thyroid lobe in only 61%^2,3^.

In studies on preoperative imaging of parathyroid adenoma, we and other authors focused on correct lateralization of the parathyroid adenoma since lateralization allows for minimally invasive parathyroidectomy and lower surgical complication rates. Ultrasound, [^99m^Tc]pertechnetate/MIBI SPECT/CT, 4D-CT, MRI and more recently [^18^F]FCH PET/CT have been shown useful for preoperative lateralization^4^. Several more techniques are described in new parathyroid imaging guidelines: [^11^C]methionine, [^11^C]choline, [^18^F]FCH PET/MR and [^18^F]FCH PET/4D CT^5^. Besides lateralization, the precise localization of the adenoma is of additional importance, since it may affect the choice of peroperative positioning, anaesthesia, and incision.

In follow-up clinics after surgery of parathyroid adenoma patients, one of the authors (A.V.d.B.) was struck by a recurrent finding: adenomas that were correctly lateralized by preoperative imaging and were peroperatively found in superior locations had been labelled as inferior by preoperative ultrasound and/or other imaging. The present study was set up as part of a larger retrospective review of surgically treated parathyroid adenomas^4^ to specifically address the question of how well the imaging techniques are able to distinguish upper from lower adenomas.

## Methods

### Patients

In this retrospective study, patients who underwent parathyroid surgery over 6 years from 01-01-2014 to 31-12-2019 in AZ Sint-Jan, Bruges, Belgium were identified. The study cohort has been described previously^4^. Patients with a diagnosis of primary hyperparathyroidism with one or multiple adenomas were included. Exclusion criteria were secondary/tertiary hyperparathyroidism (n = 54), concurrent head-neck malignancies (n = 4), MEN syndrome (n = 3) and missing patient record (n = 3). A total of 104 patients were included. One further patient with a unilateral double adenoma was excluded from this sub-analysis. Patient records were reviewed. Information about the preoperative investigations that were used for diagnosis and surgical planning, type of surgery and histology were collected from each file.

### Preoperative imaging modalities

Patients in our study cohort were referred for parathyroidectomy by three on-site and four external endocrinologists. Localization of the enlarged/hyperfunctioning gland was preoperatively assessed using neck ultrasound, [^99m^Tc]pertechnetate/MIBI SPECT/CT, and in some cases MRI, [^18^F]FCH PET/CT and/or 4D-CT, according to clinical need and availability. Most ultrasound studies were performed by one of our centre’s experienced endocrinologists using Aixplorer Supersonic Image (Aix-en-Provence, France). Parathyroid scintigraphy was performed in our centre for the majority of patients, using a dual tracer (111 MBq [^99m^Tc]pertechnetate, 740 MBq [^99m^Tc]Sestamibi) technique with SPECT/CT on a SymbiaT16 (Siemens, Erlangen, Germany). In four patients evaluated elsewhere, no hybrid SPECT/CT was performed. [^18^F]FCH PET/CT was performed at the University Hospital Ghent on a Siemens Biograph mCT 20 Flow PET/CT scanner (Siemens, Erlangen, Germany) with [18F]labelled fluoro-methyl-choline as a tracer, at an activity of 5 MBq/kg. Scanning was done from head to diaphragm to cover possible ectopic localizations. Information on ultrasound findings was routinely withheld from nuclear medicine physicians to obtain unbiased reports.

From the reports of these imaging studies, all by experienced imaging specialists, the localization of the enlarged/hyperfunctioning gland was described as right superior, right inferior, left superior, left inferior, bilateral, none or ectopic. Superior and inferior positions had been defined relative to the thyroid. In cases where the imager’s report included descriptive information only, enlarged/hyperfunctioning glands visualized close to the upper and middle portion of the thyroid lobes were classified as imaging-based ‘superior’. Enlarged/hyperfunctioning glands visualized close to the lower third of the thyroid lobe as well as those visualized under the thyroid were classified as imaging-based ‘inferior’.

### Surgery, histopathology & follow-up

Parathyroidectomy was performed by one of two experienced ENT head and neck surgeons (C.D. and T.V.). If the adenoma was localized preoperatively, surgery was performed through an incision of 2.5–3 cm or less. If the parathyroid adenoma was not found at the side predicted by imaging, the minimally invasive surgery was converted to a neck exploration. When no preoperative localization was available, a bilateral neck exploration was planned. The localization (originating from the right superior, right inferior, left superior or left inferior parathyroid) was determined perioperatively. The upper and lower parathyroid glands may be very close to one another on a craniocaudal axis, but the typical clue consistently used was the position of the parathyroid glands and the eventual adenoma relative to the inferior laryngeal nerve (RLN). The upper parathyroid glands typically are dorsal to the plane of the RLN whereas the lower parathyroid glands are ventral to the plane of the RLN, and upon adenomatous enlargement, the migration paths of superior and inferior gland adenomas tend to respect this plane^6^.

Once found and resected, a frozen section confirmed parathyroid tissue and perioperative PTH-level monitoring confirmed successful adenoma removal. Surgical findings with histopathologic confirmation of hyperfunctioning parathyroids were used as the standard against which to judge the imaging techniques. Patients were followed up by their referring endocrinologist.

### Statistical methods

All statistical analyses were performed in R version 4.0.1^7^. The chi-square test was used to compare the distribution of parathyroid adenomas over the right and left upper and lower positions in the study samples for the three imaging modalities. Fisher’s test was used to compare error rates. 95% confidence intervals (CI) of proportions were calculated according to Clopper-Pearson. P-values less than 0.05 were considered significant.

### Ethical considerations

This retrospective study was approved by the local ethics committee of AZ Sint-Jan Brugge-Oostende (Approval number 2579). All research was performed by relevant guidelines. For this study, informed consent has been waived by the AZ Sint-Jan Brugge-Oostende ethics committee.

## Results

The characteristics of the population studied have been reported in full elsewhere, patient demographics are shown in Table 1^4^. Out of 104 patients in that report, two in whom no definite adenoma localization was found, a further two with ectopic localizations and one patient with two adenomas on the right side have been excluded from the present analysis, leaving 99 patients (26 men and 73 women). The remaining sample included four patients with bilateral adenomas, for a total of 103 adenomas.

**Table 1:** Patient demographics.

Patients	104
Adenomas^a^	107
Single	95
Double	6
Size, mm (n = 91)^b^, mean (± SD)	18 (± 9.6)
Gender (n = 104)	
Male	25% (n = 26)
Female	75% (n = 78)
Age, (years)^b^, mean (± SD)	58 (± 13)
Pre-operative serum PTH, ng/L^c^, mean (range)	138 (24–952)
Pre-operative serum corrected Ca, mmol/L, mean (± SD)	2.81 (± 0.22)
Surgical procedure	104
Minimal invasive parathyroidectomy	68 (65%)
Neck exploration	36 (35%)

^a^In two patients no definitive adenoma localization was confirmed.

^b^Mean ± standard deviation.

^c^Mean (range).

Seventeen adenomas originated from the upper left, 32 from the lower left, 30 from the lower right and 24 from the upper right parathyroid. One hundred and two adenomas have been evaluated by ultrasound, 96 by SPECT(/CT) and 30 by PET. For PET, one (female) patient with a right upper adenoma was not taken into consideration because PET indicated a double adenoma to the right, precluding evaluation of possible upper/lower misclassification. Ultrasound reports were ambiguous as to upper or lower locations in seven patients (8% of the study sample), SPECT reports in one patient with bilateral adenoma (1% of the study sample), and PET reports in seven patients (including the same one with bilateral adenoma; 25% of the study sample). Table 2 gives the distribution of the adenomas over the four regions for each of the imaging modalities; no significant differences in the distribution of adenomas over the regions were observed over the three imaging modalities (p = 0.91 by chi-square test). For each definite surgical location, the findings of the several imaging modalities are shown with the number of adenomas to be identified, the number identified and the percentage misclassification or error rate (upper versus lower). The supplementary Table 1 gives the same information but expresses the error rate as a percentage of all parathyroid lesions surgically found in a given region whereas in Table 2 the denominator is the total number of lesions detected on the correct side. As can be seen from Table 2, the proportion of adenomas incorrectly assigned to the other location on the same side for each imaging modality was not different between the left and right sides, so we grouped both sides for analysis. Ten per cent of parathyroid adenomas surgically identified in the inferior regions and detected by ultrasound were classified as superior by this technique. Fourty-eight per cent of surgically identified superior parathyroid adenomas detected by ultrasound were called inferior. The respective error rates for [^99m^Tc]pertechnetate/MIBI SPECT/CT were 3 and 55%, and for [^18^F]FCH PET/CT 17 versus 36%. For both ultrasound and SPECT/CT upper adenomas were taken for lower ones more frequently than lower adenomas were mistaken for upper ones (p for the difference in error rate = 0.0003 for ultrasound and = 0.00001 for SPECT, by Fisher’s exact test). For PET/CT, the same trend was observed, but due to the much smaller sample, the difference did not reach statistical significance (p = 0.26 by Fisher’s exact test). Conclusions were similar when the analysis was broadened to all adenomas, including those that were not detected, instead of limited to adenomas being correctly lateralized (supplementary Table 1).

**Table 2:** Percentage of parathyroid lesions identified but misclassified according to the location of the lesion and imaging technique.

Surgical localization	Total number of adenomas among 99 patients	Ultrasound		[^99m^Tc]pertechnetate/MIBI		[^18^F]FCH PET/CT	
		Identified (%)	Misclassified (%)	Identified (%)	Misclassified (%)	Identified (%)	Misclassified (%)
Upper left	17	12/17 (70.6)	5/12 (41.7)	8/16 (50.0)	3/8 (37.5)	6/7 (85.7)	2/6 (33.3)
Lower left	32	26/32 (81.3)	3/26 (11.5)	19/30 (63.3)	0/19 (0.0)	5/6 (83.3)	1/5 (20.0)
Lower right	30	22/29 (75.9)	2/22 (9.1)	16/29 (55.2)	1/16 (6.3)	7/8 (87.5)	1/7 (14.3)
Upper right	24	17/24 (70.8)	9/17 (52.9)	12/21 (57.1)	8/12 (66.7)	8/8 (100.0)	3/8 (37.5)
Upper	41	29/41 (70.7)	14/29 (48.3)	20/37 (54.1)	11/20 (55.0)	14/15 (93.3)	5/14 (35.7)
CI		54.5–83.9	29.4–67.5	36.9–70.5	31.5–76.9	68.1–99.8	12.8–64.9
Lower	62	48/61 (78.7)	5/48 (10.4)	35/59 (59.3)	1/35 (2.9)	12/14 (85.7)	2/12 (16.7)
CI		66.3–88.1	3.5–22.7	45.7–71.9	0.1–15.0	57.2–98.2	2.1–48.4
P upper vs lower		NS	0.00029	NS	0.000014	NS	0.26
Total	103	77/102 (75.5)	19/77 (24.7)	55/96 (57.3)	12/55 (21.8)	26/29 (89.7)	7/26 (26.9)
CI		66.0–83.5	15.6–35.8	46.8–67.3	11.8–35.0	72.6–97.8	11.6–47.8

Misclassified = parathyroid lesions assigned to the correct side (right or left) but wrongly assigned to the upper/lower region.

No statistical differences were found in detection rates between the 4 locations.

*CI* 95% confidence interval.

**Figure 1:**
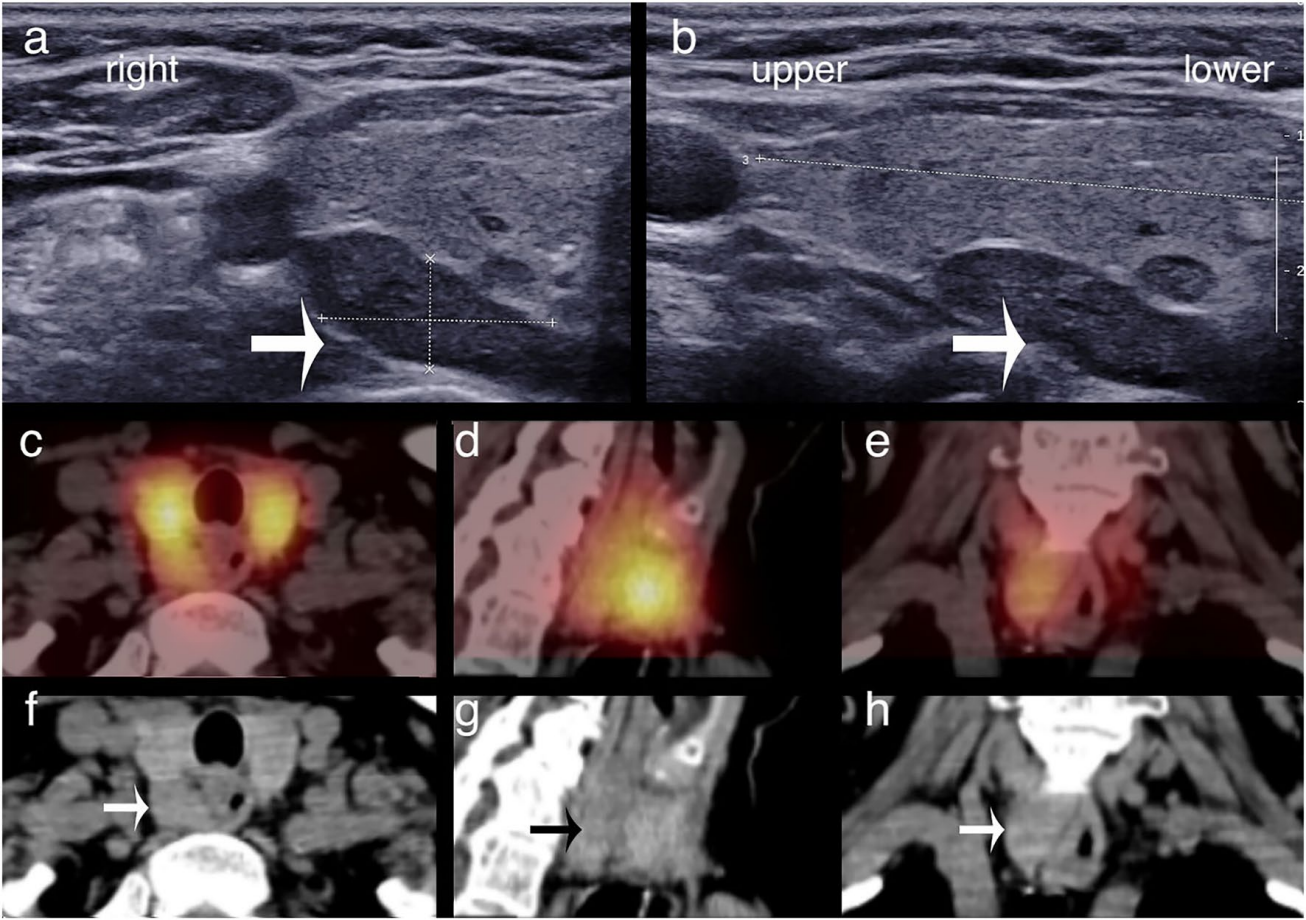
Upper parathyroid adenomas correctly localised by ultrasound and [^99m^Tc]pertechnetate/MIBI SPECT/CT. (**a,b**) Transverse and longitudinal ultrasound sections. (**c–e**) Fused [^99m^Tc]pertechnetate/MIBI SPECT/CT axial, sagittal and coronal slices and (**f–h**) present the corresponding CT slices all in the same patient with an upper right adenoma. Both ultrasound and [^99m^Tc]pertechnetate/MIBI SPECT/CT correctly identified the adenoma (arrows on ultrasound and CT images) as an upper adenoma, adjoining the middle third of the thyroid lobe.

**Figure 2:**
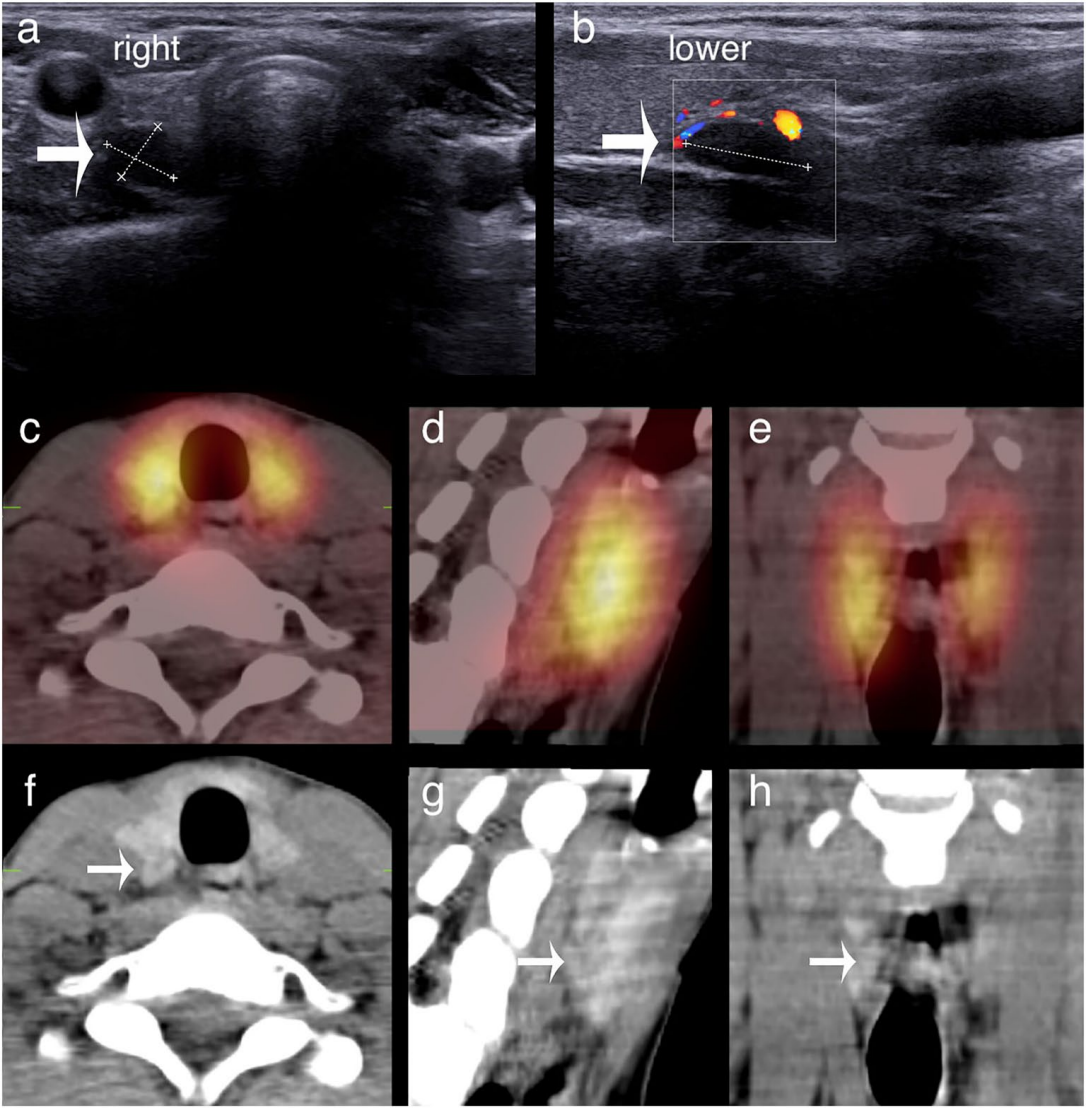
Upper parathyroid adenoma localised inferiorly by ultrasound and [^99m^Tc]pertechnetate/MIBI SPECT/CT. (**a,b**) Transverse and longitudinal ultrasound sections. In (**b**), only the lower part of the thyroid is seen. (**c–e**) Fused [^99m^Tc]pertechnetate/MIBI SPECT/CT axial, sagittal and coronal slices. (**f–h**) Corresponding CT slices. The adenoma (arrows) is seen adjacent to the inferior third of the right thyroid lobe but was surgically revealed to be an upper adenoma.

**Figure 3:**
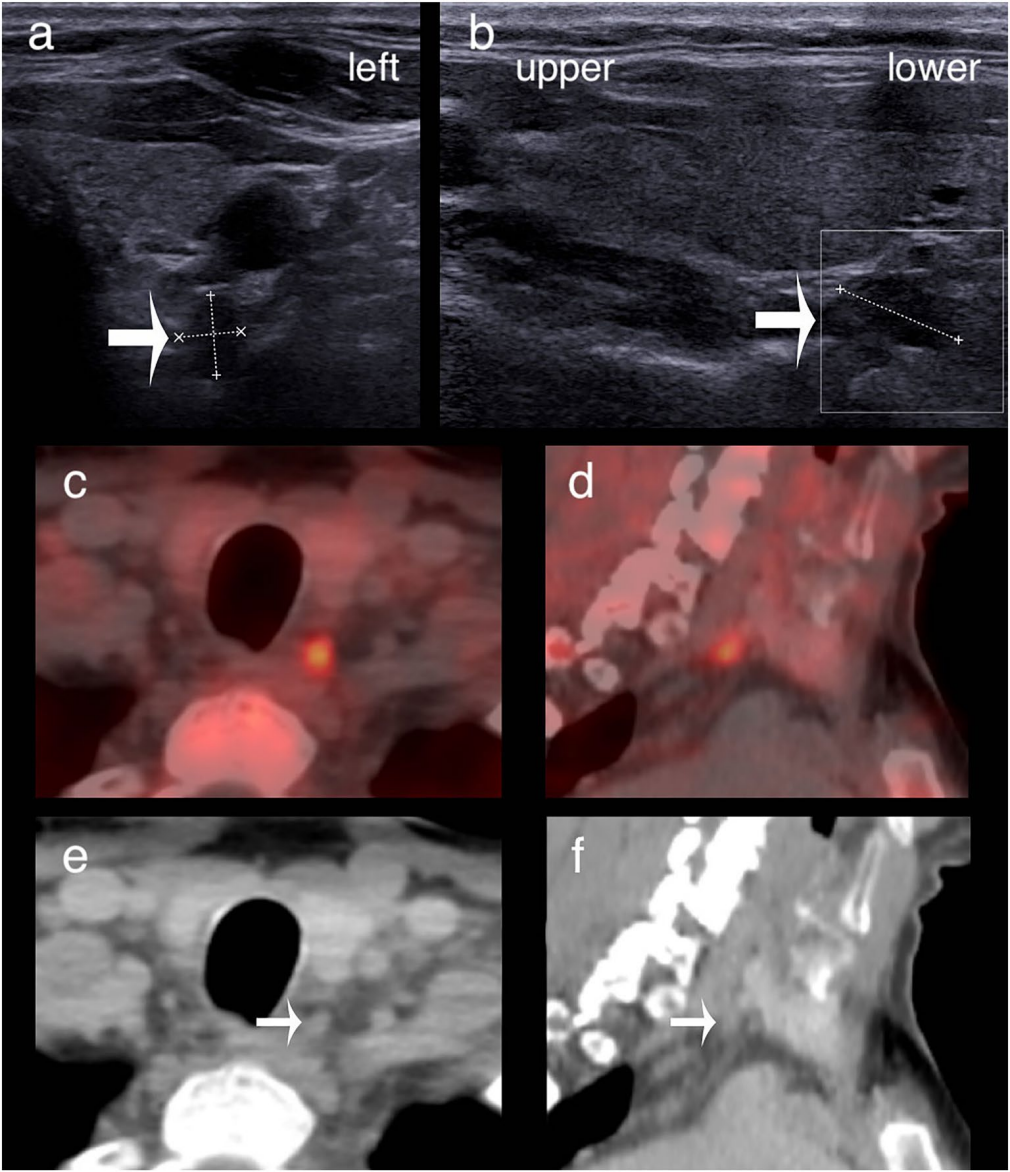
Upper parathyroid adenoma localised inferiorly by ultrasound and [^18^F]FCH PET/CT. (**a,b**) Transverse and longitudinal ultrasound sections. (**c,d**) Fused [^18^F]FCH PET/CT axial and sagittal slices. (**f**) Corresponding CT slices. The adenoma (arrows) is seen posteriorly to the inferior third of the left thyroid lobe but was surgically revealed to be an upper adenoma.

Figures 1, 2 and 3 illustrate some typical imaging findings.

## Discussion

The American Head and Neck Society Endocrine Section emphasizes precision preoperative localization to avoid unnecessary repeat operations^8^. Multimodal imaging is used to predict lateralization and the exact location of the parathyroid adenoma, allowing for minimally invasive surgery. Correct lateralization of the parathyroid adenoma is the most critical element in this respect, but imaging reports typically contain additional information on the estimated origin of the parathyroid adenoma, either superior or inferior.

In follow-up clinics of primary hyperparathyroidism patients, we were struck by a recurrent finding/error: a parathyroid adenoma designated as ‘inferior’ parathyroid adenoma based on ultrasound and/or other imaging was correctly lateralized, nevertheless identified as a size fitting superior parathyroid adenoma based on surgical findings.

In the present retrospective study, surgical findings with histopathologic confirmation of hyperfunctioning parathyroids were used as the standard against which to judge the imaging techniques. Across techniques, about one-third of superior adenomas (and up to one half of those detected by a particular imaging technique) were called inferior by imaging whereas only a small proportion of inferior adenomas had been called superior by imaging.

Our findings seem to be in accordance with those described in the chapter in the ‘Atlas of parathyroid imaging and pathology’ devoted to the anatomy of parathyroid adenoma^1^. The section on the right *superior* adenoma contains four examples, in which imaging identified the adenoma adjacent to the *mid to lower pole, at the lower pole, at the right middle pole and the level of the lower half of the right lobe* respectively. Of the four left *superior* adenomas, three were located posterior to the superior pole of the thyroid lobe and one at the level of the lower half of the left thyroid gland. Imaging localized seven out of eight inferior adenomas inferior to the inferior pole, and one subjacent to the lower pole of the thyroid.

One of the causes for upper/lower mispositioning by imaging undoubtedly relates to the fact that while the position of the parathyroid gland relative to the juxtaposition of the recurrent laryngeal nerve and the inferior thyroid artery is crucial in determining the origin of the parathyroid gland (inferior parathyroid glands typically being anterior and superior glands posterior to this juxtaposition), the recurrent laryngeal nerve cannot be visualized by any imaging modality. Moreover, imaging reporting may be unduly influenced by schematic textbook pictures showing superior and inferior parathyroid glands adjacent to the upper and lower pole of the thyroid, respectively^9–11^. It would appear from our study that the position of the parathyroid gland relative to the thyroid in the supine position is frequently not a correct guide to the origin of the gland.

Perrier et al. proposed and clinically validated a nomenclature which takes into account the more complex anatomy^12–15^. More extensive distribution of Perrier’s accurate parathyroid adenoma figure might contribute to a better knowledge by physicians involved in preoperative imaging. Our study confirms that a simple dichotomy between superior and inferior parathyroid adenomas, apart from being less informative than the Perrier classification, may also be misleading, in particular in the case of superior adenomas of which about one-third are mistaken for inferior ones.

The retrospective nature of the present study may constitute a weakness. In this real-life study, the analysis relied on the imaging reports retrieved from the patient’s record. However, these had all been produced by board-certified imaging specialists consistently judging the relative position of the parathyroid lesion to the thyroid. Apart from mistakes, it seems unlikely that re-reading by a central reviewer would have changed much. In a small number of patients, imaging reports were ambiguous as to the upper or lower origin of the enlarged parathyroid glands. However, in those patients, we assigned the enlarged glands to the upper location, going against our study hypothesis and thereby strengthening our findings. A second limitation is our study's low detection rate of [^99m^Tc]pertechnetate/MIBI SPECT/CT. Meta-analyses by Treglia and Wong found an estimated pooled sensitivity of 0.86 and 0.88 respectively on a per-patient basis (and based on publications between 2003 and 2015)^16,17^. Some recent large series with patient characteristics similar to ours show similarly lower sensitivities^18,19^. An evolution towards series with lower mean calcium, with a higher proportion of normocalcemic hyperparathyroidism, might contribute to lower sensitivities. Likewise, the addition of [^18^F]FCH PET/CT facilitated surgery in our patients with negative [^99m^Tc]pertechnetate/MIBI SPECT/CT and might somewhat lower the [^99m^Tc]pertechnetate/MIBI SPECT/CT detection rate in a surgical group enriched with [^99m^Tc]pertechnetate/MIBI SPECT/CT-negative patients as compared to patient series 2003–2015.

In conclusion, we report that ultrasound, [^99m^Tc]pertechnetate/MIBI SPECT/CT and [^18^F]FCH PET/CT frequently mistake superior parathyroid adenomas for inferior ones. Awareness that a significant proportion of upper parathyroid adenoma may be adjacent to the lower third of the thyroid lobe may contribute to more precise preoperative localization of the parathyroid adenoma.

## Supplementary Information

Supplementary Table 1.

## Data Availability

The datasets generated and analysed during the current study are available from the corresponding author upon reasonable request.
