# Supplementary material for: Preoperative imaging for hyperparathyroidism often takes upper parathyroid adenomas for lower adenomas
Source: Sci Rep. 2023 May 9;13:7568. doi: 10.1038/s41598-023-32707-0 (PMC10169799; doi:10.1038/s41598-023-32707-0)
Supplement: Supplementary file 1 — Supplementary Table 1. [file 41598_2023_32707_MOESM1_ESM.docx]

**Supplementary table** **1**– Percentage of parathyroid lesions identified but misclassified according to the location of the lesion and imaging technique. The same data as in table 1, but misclassification is expressed as a percentage of parathyroid lesions found at surgery (not only those identified by the technique).

| **Surgical localization** | **Total number of adenomas among 99 patients** | **Ultrasound** | | **[99mTc]pertechnetate/MIBI** | | **[18F]FCH PET/CT** | |
| --- | --- | --- | --- | --- | --- | --- | --- |
|  |  | Identified (%) | Misclassified* (%) | Identified (%) | Misclassified* (%) | Identified (%) | Misclassified* (%) |
| Upper left | 17 | 12/17 (70.6) | 5/17 (29.4) | 8/16 (50.0) | 3/16 (18.8) | 6/7 (85.7) | 2/7 (28.6) |
| Lower left | 32 | 26/32 (81.3) | 3/32 (9.4) | 19/30 (63.3) | 0/30 (0.0) | 5/6 (83.3) | 1/6 (16.7) |
| Lower right | 30 | 22/29 (75.9) | 2/29 (6.9) | 16/29 (55.2) | 1/29 (3.4) | 7/8 (87.5) | 1/8 (12.5) |
| Upper right | 24 | 17/24 (70.8) | 9/24 (37.5) | 12/21 (57.1) | 8/21 (38.1) | 8/8 (100.0) | 3/8 (37.5) |
| Upper | 41 | 29/41 (70.7) | 14/41 (34.1) | 20/37 (54.1) | 11/37 (29.7) | 14/15 (93.3) | 5/15 (33.3) |
| CI |  | 54.5 – 83.9 | 20.1 – 50.6 | 36.9 -70.5 | 15.9 – 47.0 | 68.1 – 99.8 | 11.8 – 61.6 |
| Lower | 62 | 48/61 (78.7) | 5/61 (8.2) | 35/59 (59.3) | 1/59 (1.7) | 12/14 (85.7) | 2/14 (14.3) |
| CI |  | 66.3 – 88.1 | 2.7 – 18.1 | 45.7 – 71.9 | 0.0 – 9.1 | 57.2 – 98.2 | 1.8 – 42.8 |
| P upper vs lower |  | NS | 0.0012 | NS | 0.000084 | NS | 0.22 |
| Total | 103 | 77/102 (75.5) | 19/102 (18.6) | 55/96 (57.3) | 12/96 (12.5) | 26/29 (89.7) | 7/29 (24.1) |
| CI |  | 66.0 – 83.5 | 11.6 – 27.6 | 46.8 – 67.3 | 6.6 – 20.8 | 72.6 – 97.8 | 10.3 – 43.5 |

*Misclassified = parathyroid lesions assigned to the correct side (right or left) but wrongly assigned to the upper/lower region

CI = 95% confidence interval
